# A Multicenter Analysis of Patients with Bullous Pemphigoid: Clinical Characteristics and Insights into Drug-Associated Disease

**DOI:** 10.3390/ijms27125587

**Published:** 2026-06-20

**Authors:** Aleksandra Małolepsza, Aleksandra Kośny, Katarzyna Juczyńska, Joanna Czerwińska, Magdalena Jałowska, Marian Dmochowski, Aleksandra Dańczak-Pazdrowska, Agnieszka Owczarczyk-Saczonek, Irena Walecka, Cezary Kowalewski, Katarzyna Woźniak, Radosław Zajdel, Agnieszka Żebrowska

**Affiliations:** 1 Department of Dermatology and Venereology, Medical University of Lodz, 90-647 Lodz, Poland; 2 Department of Dermatology, Sexually Transmitted Diseases and Clinical Immunology, University of Warmia and Mazury in Olsztyn, 10-229 Olsztyn, Poland; 3 Department of Dermatology, Poznan University of Medical Sciences, 60-355 Poznan, Poland; 4 Dermatology Clinic, The National Medical Institute of the Ministry of Interior and Administration, 02-507 Warsaw, Poland; 5 Department of Economic and Medical Informatics, University of Lodz, 90-214 Lodz, Poland

**Keywords:** bullous pemphigoid, drug-associated bullous pemphigoid, autoimmune blistering disease, direct immunofluorescence, BP180, BP230, comorbidities, real-world study, methotrexate

## Abstract

Bullous pemphigoid (BP) is the most common autoimmune subepidermal blistering disease, predominantly affecting elderly patients with multiple comorbidities. This multicentre retrospective cohort study aimed to characterize the clinical profile, treatment patterns, and drug-associated cases of BP in a real-world setting. The study included 156 patients newly diagnosed with BP between 2020 and 2024 in four dermatology departments in Poland. Diagnosis was based on clinical features, and immunological assessment, including direct immunofluorescence (DIF), ELISA, and BIOCHIP-based indirect immunofluorescence. The mean age at diagnosis was 75.5 ± 10.9 years, and 78.85% of patients had at least one comorbidity, most commonly arterial hypertension, type 2 diabetes mellitus, and dyslipidemia. Severe pruritus was reported in 74.14% of evaluated patients. Blisters and erosions were the predominant clinical manifestations. Topical glucocorticosteroids were the most frequently used treatment, followed by systemic glucocorticosteroids and methotrexate. New drug exposure within 6 months before disease onset was identified in 14.74% of patients and was associated with a shorter time to diagnosis. Drug-associated cases showed lower BP180 ELISA positivity, although this did not remain significant after correction for multiple testing. These findings highlight the clinical complexity of BP and the importance of medication review and direct immunofluorescence in diagnostic evaluation.

## 1. Introduction

Bullous pemphigoid (BP) is the most common autoimmune subepidermal blistering disease, predominantly affecting the elderly population [1]. The disease is characterized by the presence of autoantibodies directed against components of the dermal–epidermal junction, BP180 and BP230, which are essential for epidermal adhesion [2]. Importantly, in the case of BP180, autoantibodies are primarily directed against the immunodominant non-collagenous NC16A domain [3,4]. Binding of these autoantibodies activates the complement cascade and recruits inflammatory cells, particularly eosinophils and neutrophils, leading to degradation of the basement membrane zone and subsequent blister formation. Notably, complement-independent mechanisms have also been described, highlighting the complexity of BP pathogenesis [5,6,7].

In recent years, the incidence of BP has been steadily increasing [8,9]. This trend is largely attributed to population aging, as well as the expanding use of novel drugs, medical procedures and other triggers, as potential BP inductors [10,11]. Notably, several drug classes—including dipeptidyl peptidase-4 (DPP-4) inhibitors and immune checkpoint inhibitors (ICIs), have been increasingly emphasized as the most strongly implicated agents in the development of drug-associated bullous pemphigoid (DABP) [12].

Accurate diagnosis of BP relies on a combination of clinical, histopathological, and immunological findings, with direct immunofluorescence (DIF) of perilesional skin remaining the gold standard. Serological assays, such as enzyme-linked immunosorbent assay (ELISA) and indirect immunofluorescence (IIF), provide valuable support by detecting circulating autoantibodies against BP antigens and may also serve as useful tools for disease monitoring [13].

Clinically, BP typically presents with tense blisters arising on erythematous or normal-looking skin, often accompanied by intense pruritus that may precede blister formation. Additionally, non-inflammatory phenotypes have also been described, characterized by minimal erythema; more specifically, cases in which the BPDAI erythema/urticaria subscore is <10 [14,15]. Management of BP remains challenging, largely due to the coexisting comorbidities, which limit therapeutic options and increase the risk of treatment-related adverse effects. Systemic and topical glucocorticosteroids remain the cornerstone of therapy, often combined with conventional immunosuppressive agents such as azathioprine, methotrexate, or mycophenolate mofetil. However, growing evidence highlights the potential role of targeted biological therapies, including anti-IgE and anti-IL-4/IL-13 agents, offering promising alternatives with improved safety profiles in selected patient populations [16].

The objective of this study was to provide a comprehensive characterization of patients with BP, with particular emphasis on clinical presentation, comorbidity burden, and therapeutic approaches in a real-world setting. Furthermore, the study aimed to explore differences in clinical phenotype and serological profiles between classical BP and drug-associated BP.

## 2. Results

**Figure 1:**
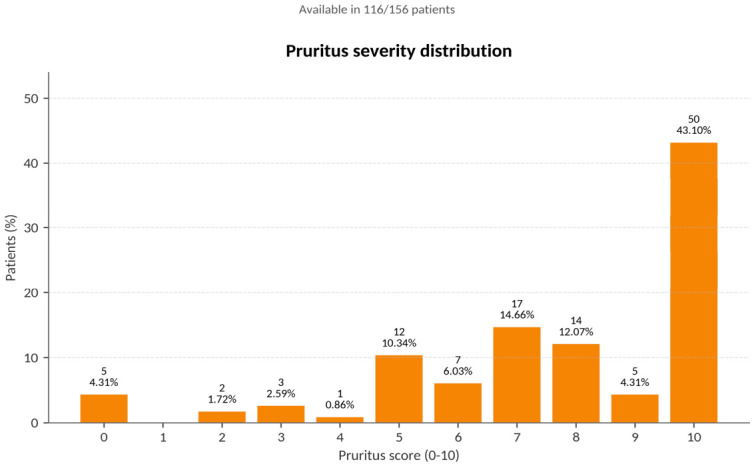
Distribution of pruritus severity in patients with bullous pemphigoid. Pruritus intensity was assessed using the Numeric Rating Scale (NRS), ranging from 0, indicating no pruritus, to 10, indicating the most severe pruritus. Values above the bars indicate the number and percentage of patients in each NRS category.

The analyzed group included 156 patients newly diagnosed with BP. The mean age at diagnosis was 75.5 ± 10.9 years. Women constituted 47.44% (n = 74) of the study population, while 52.56% (n = 82) were men. The mean time to diagnosis was 5.5 ± 6.9 months. The study population consisted predominantly of urban residents (n = 109; 69.87%), while rural residents accounted for 47 participants (30.13%). The mean time to diagnosis was 5.78 ± 7.9 months in rural patients and 5.34 ± 6.4 months in urban patients. No statistically significant difference was observed (*p* = 0.949) between these two groups. Pruritus severity was evaluated in 116 patients using the Numeric Rating Scale (NRS). Severe pruritus (NRS 7–10) was observed in 86 of them (74.14%) (Figure 1).

### 2.1. Comorbidities in Relation to Disease Severity and Clincal Phenotype

**Figure 2:**
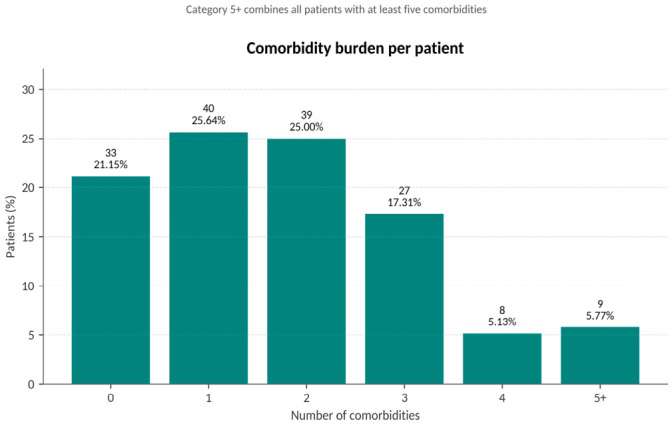
Comorbidity burden per patient in the study cohort. The category “0” indicates patients without any recorded comorbidities, whereas the category “5+” includes patients with five or more comorbid conditions. Values above the bars indicate the number and percentage of patients in each category.

The most prevalent comorbidities included arterial hypertension, type 2 diabetes mellitus, and dyslipidemia observed in 55.77%, 28.21% and 23.72% of patients, respectively. Only a minority of patients had no comorbidities (n = 33; 21.15%), while 78.85% had at least one comorbid condition. Distribution of the number of comorbidities per patient is shown in Figure 2. A comprehensive overview of all comorbidities is provided in Table 1. In the analysis including pruritus intensity, lesion morphology, mucosal involvement, and generalized disease—assessed across the most common comorbidities—arterial hypertension, type 2 diabetes mellitus, dyslipidemia, heart failure, ischemic heart disease, neurodegenerative diseases, and malignancy, several nominal exploratory signals were observed. Severe pruritus was more frequent observed in patients with type 2 diabetes mellitus than in those without diabetes (90.6% vs. 67.9%; OR 4.58, 95% CI 1.28–16.37; *p* = 0.016; q = 0.477). Generalized disease was more frequent in patients with ischemic heart disease (59.1% vs. 34.3%; OR 2.76, 95% CI 1.10–6.95; *p* = 0.033; q = 0.477). Urticarial lesions were more frequent in patients with malignancy (33.3% vs. 8.0%; OR 5.77, 95% CI 1.67 −19.90; *p* = 0.010; q = 0.477). However, these differences did not remain significant after false discovery rate (FDR) correction.

### 2.2. Diagnostic Evaluation

DIF was performed in all patients (100%), yielding positive results in 93.59% of cases. Serum analysis for the presence of antibodies using the ELISA method was performed in 114 individuals (73.08%). Anti-BP180 IgG was detected in 78.95% (95% CI 70.58–85.42) of tested patients, and anti-BP230 IgG in 24.56% (95% CI 17.57–33.21). Indirect immunofluorescence was performed in 46 patients for BP180 and in 48 patients for BP230. Reactivity against BP180 was detected in 32 participants (69.57%; 95% CI 55.19–80.92), while BP230 reactivity was observed in 12 of them (25.00%; 95% CI 14.92–38.78). DIF positivity was not significantly associated with pruritus intensity, blister formation, mucosal involvement, or generalized disease. ELISA-tested and untested patients did not differ significantly in age, time to diagnosis, pruritus intensity, or number of comorbidities. However, ELISA-tested patients received a higher number of treatment modalities than untested patients (2.18 ± 0.83 vs. 1.69 ± 0.92; *p* = 0.002; q = 0.006). In nominal analyses, male sex and excoriations were more frequent in the ELISA-tested subgroup, but these differences did not remain statistically significant after FDR correction. Patients who underwent IIF testing had a higher number of comorbidities than untested patients (2.46 ± 1.57 vs. 1.53 ± 1.42; *p* < 0.001; q < 0.001), but received fewer treatment modalities (1.69 ± 0.93 vs. 2.21 ± 0.81; *p* < 0.001; q < 0.001). Papules were more frequent in the IIF-tested subgroup than in untested patients (34.0% vs. 11.1%; OR 4.13, 95% CI 1.76–9.67; *p* = 0.001; q = 0.029). Age, time to diagnosis, and pruritus intensity did not differ significantly between IIF-tested and untested patients.

### 2.3. Skin Lesion Morphology, Distribution, and Mucosal Involvement

Blisters were observed in 139 patients (89.10%), erosions in 127 patients (81.94%), erythematous lesions in 95 (61.29%), papules in 28 (18.06%), excoriations in 27 (17.42%), and urticarial lesions in 17 (10.97%). Lesions were most frequently located on the trunk (n = 129; 82.69%), followed by the upper and lower limbs (both n = 123; 78.85%). Involvement of the head and neck was observed in 77 patients (49.36%), while the hands and feet were affected in 74 (47.44%). Generalized disease was present in 59 patients (37.82%). Mucosal involvement most commonly affected the oral mucosa (n = 32; 20.51%), while genital mucosa involvement was observed in 10 patients (6.41%) and conjunctival involvement in 6 patients (3.85%).

### 2.4. Treatment Patterns

**Figure 3:**
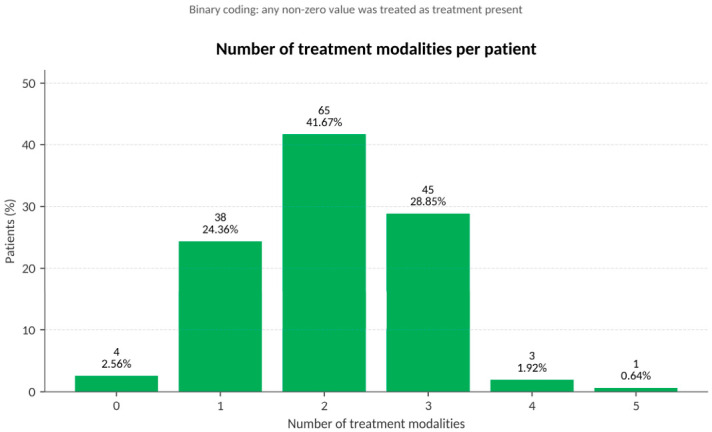
Number of treatment modalities per patient. The figure shows the distribution of patients according to the number of treatment modalities received during bullous pemphigoid management. Each treatment modality was counted as present or absent for an individual patient. Values above the bars indicate the number and percentage of patients in each category.

**Figure 4:**
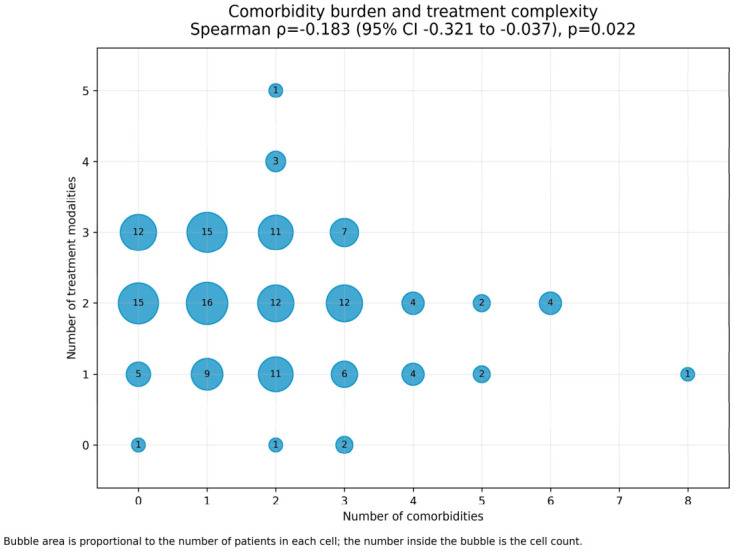
Relationship between comorbidity burden and treatment complexity in patients with bullous pemphigoid. The bubble plot shows the association between the number of comorbidities recorded per patient and the number of bullous pemphigoid treatment modalities used. Each bubble represents one combination of comorbidity count and treatment modality count. The size of the bubble is proportional to the number of patients in that category, and the number inside each bubble indicates the exact patient count.

Topical glucocorticosteroids were the most frequently used treatment (n = 123; 78.85%). Systemic glucocorticosteroids were administered in 79 patients (50.64%), and methotrexate in 51 patients (32.69%). Other treatment modalities were used in 61 patients (39.10%). Dapsone and intravenous immunoglobulins were rarely applied (n = 4; 2.56% and n = 2; 1.28%, respectively), while azathioprine was not used in this cohort. Treatment modalities used in the study cohort are presented in Table 2. Most patients received combination therapy, with the highest proportion treated with two modalities (41.67%), followed by three modalities (28.85%) and one modality (24.36%) (Figure 3). A weak inverse monotonic association was observed between the number of comorbidities and the number of bullous pemphigoid treatment modalities used per patient (Spearman rho = −0.183, 95% CI −0.321 to −0.037; *p* = 0.022) (Figure 4).

### 2.5. Clinical Characteristics of Drug-Associated Cases

Disease onset was preceded by the initiation of a new drug within 6 months prior to the appearance of skin lesions in 23 patients (14.74%). The mean age was 73.61 ± 11.36 years in the new drug group and 75.88 ± 10.82 years in the group without the new drug (*p* = 0.429). Men accounted for 47.83% of patients with new drug exposure (*p* = 0.657). No significant differences were found in age or gender distribution between the groups. Time to diagnosis was significantly shorter in patients whose lesions were preceded by new drug exposure, with a mean of 4.07 ± 7.47 months compared with 5.74 ± 6.75 months in patients without such exposure (*p* = 0.019).

The mean NRS score was 7.50 ± 2.14 in patients with new drug exposure and 7.76 ± 2.80 in those without. Median scores were 7 and 8, respectively. No statistically significant difference was observed between the groups (*p* = 0.274).

**Figure 5:**
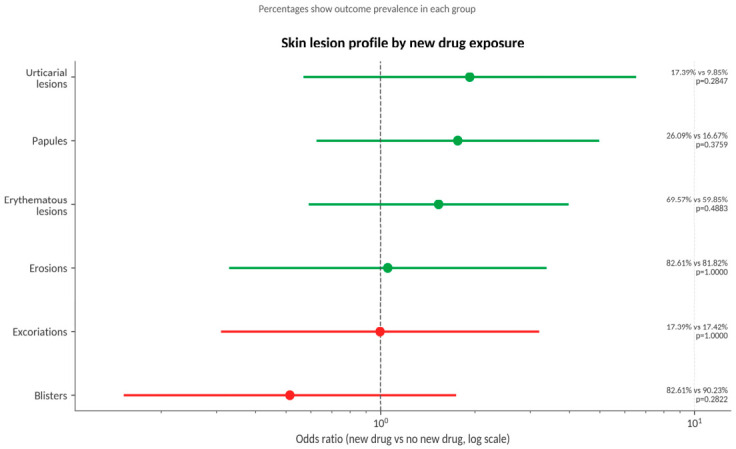
Skin lesion morphology according to new drug exposure before onset of bullos pemphigoid. The forest plot compares the prevalence of selected skin lesion types in patients with bullous pemphigoid according to whether a newly introduced drug had been recorded within 6 months before disease onset. Odds ratios are presented on a logarithmic scale. Values greater than 1 indicate higher odds of a given lesion type in patients with new drug exposure (green color), whereas values below 1 indicate lower odds in this group (red color).

Blisters were observed in 90.23% of patients without new drug exposure and in 82.61% of those with new drug exposure. No statistically significant association was found between new drug use and the presence of blisters (*p* = 0.282). Similarly, the frequency of other skin lesion types did not differ significantly between patients with and without exposure to a new drug. Erosions were present in 81.82% of patients without new drug exposure and 82.61% of those with new drug exposure (*p* = 1.000). Papules were observed in 16.67% and 26.09% of patients, respectively (*p* = 0.376), while urticarial lesions occurred in 9.85% and 17.39% (*p* = 0.285). Erythematous lesions were noted in 59.85% of patients without new drug exposure and 69.57% of those with new drug exposure (*p* = 0.488), and excoriations in 17.42% and 17.39%, respectively (*p* = 1.000) (Figure 5).

Mucosal involvement also did not differ significantly between groups. Oral mucosa involvement was observed in 22.56% of patients without new drug exposure and 8.70% of those with new drug exposure (*p* = 0.215). Conjunctival involvement occurred in 3.76% and 4.35% (*p* = 0.652), while genital mucosa involvement was noted in 6.77% and 4.35%, respectively (*p* = 0.981).

**Figure 6:**
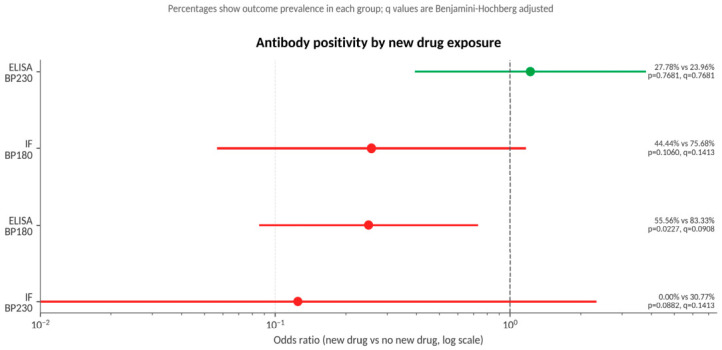
Autoantibody positivity according to new drug exposure before disease onset. The forest plot compares the frequency of positive serological and indirect immunofluorescence findings between patients whose bullous pemphigoid was preceded by exposure to a newly introduced drug within 6 months before disease onset and patients without such exposure. The analysis includes ELISA BP180, ELISA BP230, IIF BP180, and IIF BP230 positivity among tested patients. Odds ratios are presented on a logarithmic scale. Values greater than 1 indicate higher odds of antibody positivity in patients with new drug exposure (green color), whereas values below 1 indicate lower odds in this group (red color).

Anti-BP180 antibodies detected by ELISA method was observed more frequently in patients without prior exposure to a new drug (83.33%) compared with those with such exposure (55.56%). Although the difference in BP180 positivity was significant in unadjusted analysis (*p* = 0.0227), it did not remain significant after Benjamini–Hochberg correction (q = 0.0908). No statistically significant association was found between exposure to a new drug and ELISA BP230 positivity. A similar result was obtained using IIF method, with no statistically significant association observed between new drug exposure and IIF findings (Figure 6).

## 3. Discussion

Taken together, the overall findings suggest that the study cohort is representative of the typical clinical spectrum of bullous pemphigoid. The disease presentation was characterized by a predominance of blisters and erosions, with severe pruritus. Oral mucosal involvement was observed in 20.51% of patients in our cohort. This proportion is slightly higher than that reported in some European cohorts but remains broadly comparable with previously published data. Kridin and Bergman reported mucosal involvement in 17.1% of patients with BP, with oral mucosal lesions present in 13.7% of the cohort [17]. In the prospective study by Clapé et al., mucosal involvement was observed in 19% of patients, whereas Ständer et al. reported mucosal lesions in 11.4% of patients [18,19]. This variability may be explained by differences in study design, patient selection, referral patterns, diagnostic criteria, and documentation of mild oral erosions. In our case, referral bias may have contributed, as patients treated in dermatology departments, including specialized centers, may present with more extensive, atypical, or diagnostically challenging disease. At the same time, a true phenotypic difference cannot be excluded. However, the exact mechanism underlying mucosal involvement in BP remains incompletely understood.

In addition, the comorbidity profile was largely dominated by cardiovascular and metabolic conditions, which is in line with the known epidemiological characteristics of an elderly patient population [20]. Bullous pemphigoid remains a therapeutically challenging disease, largely due to frequent contraindications to commonly used treatments arising from patients’ comorbidities. Based on our findings, a substantial proportion of patients present with at least one concomitant condition, which significantly impacts therapeutic decision-making. Several nominal exploratory associations were observed between selected comorbidities and indicators of disease severity or clinical phenotype. However, none of the comorbidity–phenotype associations remained statistically significant.

Topical corticosteroids represent a relatively safe treatment option and were used in as many as 80% of patients in our cohort, underscoring their central role in disease management. However, their function is often supportive, as the daily application to the entire body surface may be difficult to implement in routine outpatient settings, particularly in elderly populations. Half of the patients in our study received systemic corticosteroids, highlighting their widespread use. Nevertheless, accumulating evidence indicates higher mortality rates and an increased risk of adverse events associated with systemic corticosteroid therapy compared with whole-body topical treatment using clobetasol propionate 0.05% [21]. Methotrexate was administered in one-third of the study population, reflecting its frequent use in real-world clinical practice. Although methotrexate is generally considered a second-line therapeutic option, the available evidence consistently demonstrates its efficacy and favourable long-term tolerability, including in patients with impaired renal function [22,23,24]. Moreover, methotrexate has been shown to exert a glucocorticoid-sparing effect, enabling a reduction in systemic corticosteroid exposure [25,26]. Emerging data further support its potential role as a therapeutic option in immune checkpoint inhibitor–induced bullous pemphigoid [26]. In addition, its use may be associated with lower mortality, likely reflecting a reduced need for prolonged systemic glucocorticoid therapy [27]. Nevertheless, some evidence suggests that methotrexate monotherapy, as compared with combination therapy with oral corticosteroids, may be associated with an increased risk of relapse [28]. Further well-designed studies directly comparing the efficacy and safety of methotrexate versus systemic corticosteroid therapy are warranted to better define its role in the management of bullous pemphigoid. A considerable proportion of patients had their treatment recorded under the “other” category. Based on routine clinical practice, this category may have included tetracyclines, particularly doxycycline, which are used as an evidence-based treatment option in selected patients with bullous pemphigoid. However, because the original retrospective data collection form did not systematically capture specific therapies within the “other” category, this assumption could not be reliably confirmed [13]. Furthermore, treatment-related data were descriptive and did not include standardized information on treatment sequence, treatment response, and relapse rates. In our cohort, patients with more comorbidities tended to receive fewer treatment modalities, suggesting that multimorbidity may limit rather than intensify therapeutic options. This is clinically plausible, as frailty, cardiovascular and metabolic comorbidities, and the risk of adverse events may reduce the feasibility of systemic treatment escalation. Thus, comorbidity burden should be considered an important factor shaping real-world therapeutic decision-making in BP.

In recent years, increasing attention has been given to drugs capable of inducing bullous pemphigoid, raising the question of how such a wide range of structurally diverse molecules can trigger the development of a single disease entity. The pathophysiology is characterized by autoantibodies directed against basement membrane proteins BP180 and BP230, but the underlying mechanisms remain incompletely understood. Some authors even distinguish two distinct entities: drug-associated BP and drug-triggered BP [29]. In the latter, the clinical course closely resembles that of classical BP, with recurrent exacerbations and remissions persisting despite withdrawal of the suspected triggering agent.

Recently, particular emphasis has been placed on the role of dipeptidyl peptidase-4 inhibitors and immune checkpoint inhibitors in the development of drug-associated bullous pemphigoid. In the context of DPP-4 inhibitors, some studies suggest a distinct clinical phenotype with less inflammatory skin manifestations, whereas others have not confirmed these differences [14,30,31,32,33,34]. These differences have been observed in studies conducted in Japan and China, whereas European studies do not emphasize this distinction. In contrast, ICI-associated cases are more commonly reported to have prolonged latency between treatment initiation and symptom onset [35,36]. Moreover, the prodromal phase, often limited to pruritus, can persist for several months. In our study, no significant differences were observed in the frequency of erythematous or urticarial lesions between patients with classical bullous pemphigoid and those with drug-associated disease. However, it should be noted that only the presence or absence of specific lesion types was assessed in our study, without evaluation of disease severity using validated instruments such as the Bullous Pemphigoid Disease Area Index (BPDAI) [37,38]. Additionally, in our cohort, patients with newly introduced drug exposure had a statistically shorter time to diagnosis than those without such exposure. However, the absolute difference was modest, and this finding should be interpreted cautiously. In routine clinical practice, the identification of a recently introduced medication may support clinical suspicion, thereby facilitating earlier management. Nevertheless, we cannot exclude other explanations, including differences in disease onset dynamics or healthcare-seeking behavior. In contrast, place of residence was not associated with differences in time to diagnosis, suggesting a comparable diagnostic pathway for patients from urban and rural areas.

There are also conflicting reports regarding seropositivity in patients with drug-associated BP. Some studies indicate lower detectability of autoantibodies [15,30,39]. On the other hand, some evidence suggests no significant differences compared with classical BP [32,34]. In our cohort, patients with newly introduced drug exposure showed a trend toward lower BP180 ELISA positivity compared with patients without such exposure. However, this difference did not remain statistically significant after correction for multiple testing and should therefore be interpreted with caution. This finding may be explained by differences in antigenic targets, as standard ELISAs are primarily designed to detect antibodies against the NC16A domain of BP180. In drug-associated BP, however, the immune response may be directed against alternative epitopes or different regions of the antigen, which could result in lower serological detectability despite the presence of active disease. In a recently conducted study by Mai et al., it was demonstrated that DPP-4 inhibitors-associated BP presents a distinct autoantibody profile compared with classical BP, as the dominant target of the immune response was the NC7–Col4 region of the BP180 protein rather than the NC16A domain, which is characteristic of classical BP [40]. They also showed that the presence of HLA-DQA1*05:05 and HLA-DQB1*03:01 alleles may predispose patients to the development of DPP-4 inhibitors-associated BP by facilitating BP180 antigen presentation and inducing autoantibodies against the NC7–Col4 region, highlighting the important role of genetic predisposition in the pathogenesis of the disease. Patients with DPP-4 inhibitors-associated BP who are positive for NC7–Col4 autoantibodies, especially those with concomitant anti-NC16A antibodies, may require more intensive treatment. On the other hand, not all patients receiving DPP-4 inhibitors who develop BP have a disease mechanism described above, which may have important implications for therapeutic decision-making and for considering the continuation of antidiabetic treatment.

The present study has several limitations. First, its retrospective design may be associated with incomplete or missing data and limits the ability to establish causal relationships. Second, the BPDAI (Bullous Pemphigoid Disease Area Index) score was not used, which restricts the objective assessment of disease severity and limits comparability with other studies. Lesion morphology was evaluated only as the presence or absence of selected lesion types, without quantitative assessment. As a result, the absence of statistically significant differences between classical and drug-associated BP should be interpreted cautiously and does not exclude clinically relevant differences in inflammatory phenotype or disease severity. In addition, treatment comparisons could not be adjusted for baseline disease severity, which limits interpretation of therapeutic patterns. Finally, drug-associated BP was defined operationally as BP preceded by the initiation of a new drug within 6 months before lesion onset; however, causality could not be established due to the retrospective design and lack of drug-specific causality assessment.

## 4. Materials and Methods

This multicentre retrospective cohort study was performed in four dermatology departments in Poland, including patients newly diagnosed with bullous pemphigoid between 2020 and 2024. The diagnosis was made in line with the European Academy of Dermatology and Venereology (EADV) criteria [13]. Drug-associated BP was defined as the initiation of a new drug within 6 months prior to the appearance of skin lesions, in accordance with these recommendations. Direct immunofluorescence was performed in all patients using perilesional skin samples. Serum samples were analyzed for circulating autoantibodies using enzyme-linked immunosorbent assay. In addition, BP180 and BP230 reactivity was assessed using BIOCHIP-based indirect immunofluorescence. Serum samples were analyzed for anti-BP180 and anti-BP230 IgG autoantibodies using commercially available ELISA kits—Anti-BP180-NC16A-4X ELISA (IgG), order no. EA 1502-4801-2 G, and Anti-BP230-CF ELISA (IgG), order no. EA 1502-4801-1 G (EUROIMMUN Medizinische Labordiagnostika AG, Lübeck, Germany)—according to the manufacturer’s instructions. The BP180 ELISA targets a tetramer of the immunodominant extracellular NC16A domain of BP180, whereas the BP230 ELISA uses a C-terminal fragment of BP230 as the antigen substrate. Clinical and laboratory data were collected retrospectively from electronic health records using a unified data collection template.

All analyses were performed using the final study dataset. Statistical analyses were performed using Statistica 13.3 (TIBCO Software Inc., Palo Alto, CA, USA). Continuous variables were summarized using the number of available observations, mean, standard deviation, 95% confidence interval for the mean, median, interquartile range, minimum, and maximum. Categorical variables were summarized as counts and percentages, with Wilson 95% confidence intervals for proportions. Binary comparisons between the new drug exposure group and no new drug exposure group were performed with Fisher exact test because the exposed group was relatively small. Odds ratios with 95% confidence intervals were calculated. Continuous variables compared between two independent groups were analysed with the Mann–Whitney U test. To complement the hypothesis tests, 95% confidence intervals for mean and median differences were estimated with nonparametric bootstrap resampling using 10,000 iterations. The comparison of age, gender, and time to diagnosis between new drug exposure group and no new drug exposure group was predefined. The comparison of skin lesion morphology included blisters, erosions, papules, urticarial lesions, erythematous lesions, and excoriations. The serologic comparison included ELISA BP180, ELISA BP230, IIF BP180, and IIF BP230 positivity among tested patients. Because multiple serologic comparisons were explored simultaneously, Benjamini–Hochberg FDR adjustment was additionally calculated as a sensitivity analysis for that family of tests. Time to diagnosis was also compared between rural and urban residents with the Mann–Whitney U test. All tests were two-sided. *p* values were reported to three decimal places. Numerical values in descriptive tables and figures were rounded to two decimal places, except for standard deviation, which was shown to three decimal places.

The study was reviewed and approved by the Bioethics Committee of the Medical University of Lodz (approval no. RNN/244/25/KE, 21 October 2025). It involved the analysis of previously collected anonymized patient data in accordance with applicable ethical standards and regulations, including the Declaration of Helsinki. There was no need to obtain patient consent to participate in the study.

## 5. Conclusions

The results of this study indicate that patients with bullous pemphigoid are most often burdened with multiple comorbidities, which may significantly limit available therapeutic options and complicate disease management. A substantial proportion of cases were identified as drug-associated bullous pemphigoid, highlighting the need for increased awareness among physicians of various specialties, as well as dermatologists. In this group of patients, a trend toward lower BP180 ELISA positivity was observed; however, this finding did not remain statistically significant and should therefore be interpreted cautiously. Therefore, direct immunofluorescence remains the gold standard and a crucial component in establishing the proper diagnosis. It should also be noted that antibodies directed against different components of the antigens typically involved in bullous pemphigoid could represent a potential source of additional information relevant to diagnostic assessment and therapeutic decision-making.

## Figures and Tables

**Table 1:** Comorbidity profile of patients with bullous pemphigoid. Comorbidities are grouped according to major clinical categories. Percentages were calculated in relation to the total study population. Since individual patients could have more than one comorbidity, categories are not mutually exclusive and percentages do not sum to 100%.

Comorbidity	N	%
**Cardiovascular diseases**		
Hypertension	87	55.77
Heart failure	31	19.87
Ischemic heart disease	22	14.10
Cerebrovascular disease	87	55.77
**Metabolic disorders**		
Type 2 diabetes mellitus	44	28.21
Dyslipdemia	37	23.72
**Neurological diseases**		
Neurodegenerative diseases	16	10.26
Epilepsy	2	1.28
**Psychiatric disorders**		
Affective disorders	8	5.13
Anxiety disorders	5	3.21
Personality disorders	4	2.51
Psychotic disorders	1	0.64
**Other**		
Malignancy	15	9.62

**Table 2:** Real-world treatment patterns in patients with bullous pemphigoid. Since some patients received combination therapy, treatment categories are not mutually exclusive and percentages do not sum to 100%.

Treatment Modality	N	%
Topical corticosteroids	123	78.85
Systemic corticosteroids	79	50.64
Methotrexate	51	32.69
Other	61	39.10
Dapsone	4	2.56
Intravenous immunoglobulins	2	1.28
Azathioprine	0	0

## Data Availability

The data included in this study are available on request from the corresponding author.

## Abbreviations

The following abbreviations are used in this manuscript:

BP	Bullous Pemphigoid
DABP	Drug-Associated Bullous Pemphigoid
DIF	Direct Immunofluorescence
IIF	Indirect Immunofluorescence
ELISA	Enzyme-linked immunosorbent assay
NC16A	Non-collagenous 16A domain
NC7-Col4	NC-7-collagenous IV region
DPP-4	Dipeptidyl peptidase-4
ICI	Immune Checkpoint Inhibitor
IgE	Immunoglobulin E
IgG	Immunoglobulin G
IL-4	Interleukin-4
IL-13	Interleukin-13
BPDAI	Bullous Pemphigoid Disease Area Index
NRS	Numeric Rating Scale
CI	Confidence Interval
OR	Odds Ratio
IQR	Interquartile Range
SD	Standard Deviation
FDR	False discovery rate
HLA	Human Leukocyte Antigen
